# Untreated early childhood caries is a potential disability: policy and programme implications for Africa

**DOI:** 10.3389/froh.2025.1546747

**Published:** 2025-05-21

**Authors:** Moréniké Oluwátóyìn Foláyan, Adeyinka Ganiyat Ishola, Olunike Rebecca Abodunrin, Nicaise Ndembi, Maha El Tantawi

**Affiliations:** ^1^ The Africa Oral Health Network (AFRONE), Alexandria University, Alexandria, Egypt; ^2^ Department of Child Dental Health, Obafemi Awolowo University, Ile Ife, Nigeria; ^3^ Department of Nursing, University of Ibadan, Ibadan, Nigeria; ^4^ Department of Epidemiology and Biostatistics, Nanjing Medical University, Jiangsu, China; ^5^ Director General's Office, International Vaccine Institute (IVI), Africa Regional Office, Kigali, Rwanda; ^6^ Division of Epidemiology and Prevention, Institute of Human Virology, University of Maryland School of Medicine, Baltimore, MD, United States; ^7^ Department of Pediatric Dentistry and Dental Public Health, Alexandria University, Alexandria, Egypt

**Keywords:** oral health, public health, primary health care, health policy, preschool child, health care integration, dental products

## Abstract

Early Childhood Caries (ECC) is a significant oral health condition that impacts children globally. This manuscript's main objective is to explore ECC's impact on children's oral health-related quality of life (OHRQoL) in Africa, and to highlight the policy and programme recommendations to eliminate untreated ECC as a public health threat in Africa. In Africa, ECC poses a significant public health challenge and has the potential to result in functional disabilities in children. A rapid review of the literature focusing on studies from Africa explored the impact of ECC on children's oral health-related quality of life. The three studies that met the eligibility criteria revealed that ECC negatively impacts multiple dimensions of life, including physical health (pain, malnutrition, chewing difficulties), psychological well-being (low self-esteem, stigma), and social functioning (peer relationships, school attendance). Advanced ECC had more pronounced effects, particularly in the symptom and psychological domains. The paper highlights the urgent need to recognise untreated ECC as a disability within public health frameworks in Africa. Policy recommendations include integrating oral health into primary healthcare systems, expanding community-based prevention programmes, incentivising the production of affordable oral health products, and developing school-based education initiatives. Strengthening oral health workforce capacity and enhancing data collection on ECC prevalence is critical for effective policy formulation and resource allocation. Recognising ECC as a potential disability underscores the need for a multi-sectoral approach to address this neglected public health priority and for prioritising actions to eliminate untreated ECC as the International Day of Persons with Disabilities (December 3) is marked.

## Introduction

Early Childhood Caries (ECC) is a debilitating disease. It is defined as one or more decayed, missing (due to caries), or filled tooth surfaces in any primary tooth in a child aged 71 months or younger (1). It is also a widespread oral disease that affects infants, toddlers, and preschoolers. The global prevalence ranges from 46.2% (2) to 48% (3). The prevalence increases with age (4) from 17% in one-year-olds to 36%, 43%, 55%, and 63% for 2, 3, 4- and 5-year-olds, respectively (5). More than 530 million children worldwide have ECC, and most ECC cases remain untreated (6). Although the disease is decreasing globally, it is increasing in Africa (7).

ECC is a disability because it is a complex and multifaceted disease that encompasses physical, mental, and social impairments (8). Traditionally, disability has been associated with visible physical limitations or chronic illnesses. However, the World Health Organization (WHO) recognises that disability can also stem from impairments in various domains. Disability is defined as a “dynamic interaction between health conditions and contextual factors.” (8) This definition highlights that a person's health condition does not solely determine disability but is also influenced by social, environmental, and personal factors (8).

This untreated burden not only compromises oral health but also has far-reaching consequences on the physical, psychological, and social development of the child, particularly in low-resource settings like Africa. Against this backdrop, the main objective of this manuscript is to explore the impact of ECC on children's oral health-related quality of life (OHRQoL) in Africa and to highlight policy and programme recommendations aimed at eliminating untreated ECC as a public health threat across the continent.

## Untreated early childhood caries and disability

Untreated ECC is associated with conditions that can cause disability. It can lead to infection, pain, abscesses (8), difficulty eating and chewing, malnutrition (9), and difficulty in sleeping (10, 11). The cumulative effect of untreated ECC on the health and well-being of the child impacts physical growth and psychological development (12). This results from the impaired masticatory function of the hippocampus, chronic inflammation, and sleep disturbances that negatively affect cognitive functioning (13). These gut microbiome disruptions due to frequent antibiotics and analgesics to treat ECC-related infections, and the elevated nutritional risk from untreated ECC, can undermine optimal brain and cognitive development in young children (13). Children with untreated ECC have higher risks of developing dental caries in adolescence (14).

Physical development in early childhood is vital for an individual's long-term health and functional ability. Poor physical development can hinder the development of essential motor skills and functional capacity, thereby increasing susceptibility to disability. Physical development encompasses gross and fine motor skills, essential for daily self-care (15). Children experiencing delays in motor skills may face challenges in mobility and coordination, limiting their ability to participate fully in age-appropriate activities (16). Poor physical development often results in increased vulnerability to infections, chronic illnesses (17), functional impairments, and developing disabilities later in life. Lack of early intervention in physical development can lead to irreversible health conditions and impairments that restrict physical independence (18, 19). These impairments may qualify as a disability when they prevent individuals from performing daily activities and meeting social expectations.

In addition, psychological development is integral to a child's ability to function and engage with their surroundings. For example, children with a positive self-image and healthy self-concept are likelier to take on challenges and persevere through difficulties (20). ECC, however, increases the risk of low self-esteem (21) and poor social interaction (12, 22). Social exclusion can also result from stigma (23). In addition, premature tooth loss caused by untreated ECC can impede speech clarity and fluency (8, 12, 24, 25), resulting in communication barriers that limit interaction with peers and engagement in educational activities, ultimately leading to social isolation (26).

Furthermore, poor psychological development poses significant risks for disability when cognitive development is compromised or communication, comprehension, and learning are affected. Cognitive impairments due to untreated ECC (27) can also negatively impact educational attainment and the development of adaptive skills, which are critical for independent living.

Children with ECC frequently report experiencing pain, difficulty with eating, and psychological distress, all of which disrupt their daily activities and overall well-being (28–30). The oral health-related disabilities resulting from ECC affect the OHRQoL and negatively impact an individual's physical, social, and psychological well-being (31). Untreated ECC consistently negatively impacts the OHRQoL of children and their families (32). The most significant impacts are observed in the symptom and psychological domains (32, 33). In addition, ECC predicts reduced OHRQoL as children age (28, 34).

## Untreated early childhood caries and disability in children in Africa

The prevalence of ECC in Africa is high, estimated to be 30% (2), with wide variations among countries on the continent (35). The burden of untreated ECC is likely to increase in Africa largely due to population growth, increasing survival rates in early ages, and a high proportion of young populations (36). Whereas the population is expected to decrease somewhat until 2,100 in Asia, Europe, and South America, it is predicted to grow significantly in Africa (37).

There are high concerns about the high risk of functional disability from untreated ECC in Africa. This is related to social, environmental, and personal factors, including access to healthcare and poor supportive family and community environments. Access to oral healthcare facilities is notably limited by several barriers and challenges, including a shortage of trained oral health professionals on the continent (38), uneven distribution of healthcare resources with a concentration in urban areas (39), and limited public funding for oral health services (40). In addition, these issues exacerbate the low prioritisation of oral health in public health policies (41, 42) and poor access to and uptake of preventive dental care.

ECC is not only a dental health issue but also a social concern. It is linked to poverty (43, 44), lower socioeconomic status (45–47), living in low- and middle-income countries (48, 49), and living in poor households that cannot afford dental care, healthy foods, or oral hygiene products (50). Thus, ECC is a social justice issue (51), and the risk of ECC progressing to a disabling condition increases when families lack the resources, knowledge, or access to support systems.

The burden of ECC is also higher in communities where caregivers are not educated on prevention or access to services like fluoride treatment or affordable dental check-ups (52, 53) and in areas where dental care is less prioritised (54). There is limited focus on community programmes that improve access to oral care in Africa (55, 56). The risk factors associated with a higher risk of ECC are also associated with the risk of worse OHRQoL outcomes for ECC in Africa (57).

We conducted a rapid scoping review of the literature for studies on the associations between ECC and the OHRQoL of children in Africa. A search for relevant literature was conducted on the Web of Science, PubMed, and CINAHL databases. Studies were included if they included information about dental caries and OHRQoL, if the target group was children younger than 6 years of age, or if information about them could be extracted, and if they lived in the 54 African Union member states. Studies were included if they were cross-sectional, cohort, case-control, randomised controlled trials, quasi-experimental studies, systematic reviews, or policy analyses. Opinion pieces, editorials, studies lacking primary data on the association between ECC and OHRQoL, and studies not published in English were excluded.

Our search strategy incorporated the following terms: [“early childhood caries” OR ECC OR “dental caries” OR “dental decay” OR “dental cavity” OR “dental cavities” OR “tooth decay” OR (Infant AND “Dental Cavity”) OR (child* AND caries)] AND [“Oral health-related quality of life” OR OHRQL OR (“oral health” AND quality of life) OR (“dental health” AND quality of life) OR OHRQoL] AND Africa.

The review was conducted by two independent reviewers (AGI and ORA) with conflict resolution by a third reviewer (MOF). Rayyan was utilized to manage and screen articles. Standardized forms captured details on study location, study objective, study design, sample size, and study findings.

We identified 75 studies—PubMed (31), Web of Science (36), and CINALH (8)—of which 22 were excluded for duplication. Of the 53 titles/abstracts screened, 40 were excluded for reasons ranging from study participants were not children (21), to study location was outside the scope (8), and study did not report OHRQoL (11). Of the 13 articles sought, 10 were excluded for reasons ranging from the study not focused on Africa (2), to review articles (2), study focused on older ages (4), and ECC not reported (2). Three studies met the eligibility criteria.

The summary of the publications is highlighted in Table 1. The three studies were cross-sectional, conducted in East Africa, enrolled 1,723 children, and were conducted between 2013 and 2020. Like other studies, they indicated that untreated ECC affects multiple dimensions of life, including physical health (pain, discomfort, and dietary restrictions) (58), psychological well-being (stigma and self-esteem) (59), and social functioning (peer relationships and school attendance) (58, 59), in addition to family impacts (60). Although there are few studies, these regional findings may be generalisable to the rest of Africa because the risk factors for ECC and its non-treatment in East Africa are similar to those in most of the other regions of the continent (61), thereby supporting the applicability of these findings to a continental scale although contextual differences are acknowledged.

**Table 1:** Studies reporting on early childhood caries and oral health quality of life.

Author and year of publication	Study location	Study objective	Study design	Sample	Study findings
Masiga and M'Imunya, 2013 (58)	Kenya	To determine the prevalence of dental caries and its impact on QoL among HIV-infected children in Kenya	Cross-sectional	200	Children with high dmft had negative impacts on their appearance, chewing, biting hard foods, and missing school on account of toothache and discomfort.
Masumo et al, 2020 (59)	Tanzania	To assess the prevalence of dental caries and its impacts on the OHRQoL in a sample of preschool children in Kisarawe	Cross-sectional	1,106	Preschool children who replied “yes” to “Do your teeth hurt you now?” and “Do kids make fun of your teeth?” were likelier to have a decayed tooth.
Birungi et al, 2016 (60)	Uganda	To investigate whether children's and caretakers’ caries experience is associated with oral health-related quality of life (OHRQoL) in children and their families	Cross-sectional	417	Children's caries experience was associated with worse OHRQoL in children and their families.

The findings of the rapid review indicate that children with high dmft experience significant challenges, including negative impacts on appearance, chewing difficulties, and school absenteeism due to toothache and discomfort. These limitations interfere with essential daily activities such as eating and learning (58), contributing to malnutrition, weight loss, and impaired physical development—hallmarks of functional disability (62). Dental pain further exacerbates nutritional deficiencies, potentially delaying both physical and cognitive growth (63), while missed school days hinder educational attainment, reinforcing long-term disadvantages (64). Beyond physical effects, untreated ECC leads to low self-esteem, social anxiety, and psychological distress (65). Visible decay, pain, and tooth loss from ECC can result in peer stigma and bullying (66) and isolation (67), negatively affecting mental health and social interactions. This aligns with the WHO's definition of disability, which includes impairments in social participation and psychological well-being (7).

In addition, ECC's negative impact on children and their families reflects the broader interaction between health conditions and environmental barriers, worsening OHRQoL. Chronic pain and malnutrition can disrupt sleep, impair cognitive function, and weaken immune responses, increasing susceptibility to illness (68). These cumulative effects restrict a child's ability to function and participate fully in society, reinforcing ECC's classification as a disabling condition.

## Policy and programme implications to reduce the risk of disability from ECC in Africa

Policy development is a crucial function of public health to enhance population health (69). Health policies guide governments to desired health outcomes and outline the resources needed to achieve these outcomes (70). Recognising ECC as a potential disability for children in Africa calls for comprehensive health policy reform. Such reform includes expanding access to oral healthcare, enhancing public education and awareness, and increasing funding and resources for children's oral health programmes. Policy modifiable factors can address the inequity driving ECC (71). The policies should use a multi-sectoral approach by embedding oral health into existing healthcare and community systems and creating supportive environments to address systemic barriers and enhance community engagement.

Integrate Oral Health into Primary Healthcare Systems: The call to integrate oral health into primary healthcare (PHC) was made in the 1,978 Declaration of Alma-Ata, which emphasised the importance of PHC in achieving “Health for All.” (72). The declaration underscored the need for accessible, community-based preventive and curative services. Since then, the integration is still an important healthcare pathway (73).

In many parts of Africa, PHC is often the first—and sometimes only—point of contact for healthcare (74), making it an effective approach to address ECC. This entails the inclusion of basic oral health screenings in routine pediatric visits at PHC centres, establishing guidelines for ECC risk assessment and preventive counselling, and fluoride application. It also requires training PHC personnel, including community health workers, to detect ECC, educate parents on preventive practices, and apply simple and effective interventions, such as fluoride varnish.

The concept of PHC, however, has to expand beyond Western orthodox practices because child care often occurs through diverse, culturally rooted pathways (75). Traditional healers, family networks, and community leaders play significant roles in children's health in many African communities. Expanding PHC to embrace these local systems can enhance access to child oral health services, promote preventive practices, improve outcomes, reach underserved populations, and increase healthcare utilisation, thus providing a comprehensive care model for ECC.

### Expand community-based oral health programmes

Community-based programmes help reach populations with limited access to formal dental services. These programmes leverage community members to promote oral health awareness, prevention, and early intervention. It is important to support the development of community health programmes dedicated to ECC prevention by establishing partnerships with local NGOs, religious institutions, and schools. Mobile dental clinics can also provide preventive and basic curative services by visiting remote areas and offering screenings, fluoride treatments, and referrals.

Most community-based health programmes are in South Asian and Sub-Saharan African countries (76) and are run by community health workers (CHWs). However, there is scarce evidence about using CHWs for oral health programmes (77). Evidence is needed on the effectiveness and cost-effectiveness of engaging CHWs in implementing oral health programmes in rural and underserved African areas with a workforce shortage to deliver oral health care.

### Local production and distribution of affordable oral health products

Access to affordable toothbrushes, fluoride toothpaste, and other oral hygiene products remains limited in many African countries. The affordability and availability of oral health products may be improved by policies that promote local production through tax breaks and subsidies for local manufacturers, public-private partnerships, and the use of corporate social responsibility for communities that host the business.

The African Union (AU) and Africa Centres for Disease Control and Prevention (Africa CDC) have rightly prioritised the local production of vaccines and therapies to bolster continental resilience in times of health crises, where rapid access to treatments and vaccines can save lives (78). However, this prioritisation often overlooks non-crisis health issues like oral health, which, although not immediately life-threatening, lead to long-term disability, economic burdens, and reduced quality of life across the continent. The absence of local production of oral hygiene products perpetuates disparities, as many communities face limited access to basic dental care, which has implications for overall health (79). Failing to address oral health thus represents a missed opportunity for holistic disease prevention across Africa, limiting progress towards comprehensive public health. Policies should support the local production of essential oral health products to reduce the prevalence of ECC and other oral diseases and enhance overall health equity across Africa.

### Implement school-based oral health education and preventive programmes

Schools provide a powerful platform for reaching young children by fostering early positive habits (80). Developing national oral health programmes focusing on daily oral hygiene, healthy eating, ECC risk assessment, and prevention can reduce ECC prevalence (81). In addition, establishing regular dental screenings in schools, especially for early childhood, like the “Toothbrushing with Fluoride” programme in Thailand (82), can make a difference. This programme provided fluoride toothpaste and toothbrushes for children to use at school daily, reinforcing healthy habits and reducing decay rates over time.

School-based oral health education and preventive programmes in Africa would, however, face challenges due to insufficient funding, shortage of skilled workforce, limited parental involvement (83), poor oral health literacy, and priority given to the primary dentition (84) and the large number of preschool children who are not in school. Currently, 19.7% of children in Africa are not attending school, and 30% of the 250 million out-of-school children globally are in Sub-Saharan Africa (85). Addressing these factors using a multi-sectoral approach to community development would enable the implementation of these programmes.

### Develop public awareness campaigns

Awareness of ECC and its impact is limited in many African communities. Public campaigns can increase awareness, shift social norms about oral health, and motivate families to seek early care. Mass media, including radio, television, and social media, can reach a broad audience. However, the messages must be tailored to local cultural beliefs and practices around oral health.

### Strengthen oral health workforce capacity

Addressing ECC requires increasing the number and distribution of trained oral health professionals across the continent. Currently, most countries in the WHO African Region do not have enough oral health workforce to address their population's oral health needs, and they will still not have enough by 2030. Task shifting and training other healthcare professionals to provide oral health and ECC-specific services can help address this shortage (86). This includes general health practitioners, paediatricians, nurses, and mid-level providers. By empowering a broader range of healthcare providers with ECC prevention and management skills, early intervention and preventative care at the community level may be possible, especially in underserved and remote areas where dentists are scarce.

### Utilise data to inform policies and programmes

Data on ECC prevalence, risk factors, and impact across Africa is limited. Expanding surveillance systems and conducting regular oral health assessments can inform targeted interventions and help allocate resources where they are most needed. Few African countries, like Egypt, Namibia, and South Africa, have been reported to have collected routine surveillance data on ECC (87). Monitoring ECC requires funding research to understand ECC trends and establishing a national ECC registry to monitor intervention responsiveness. Routine ECC surveillance can also be integrated into global surveys such as the Demographic Health Survey, conducted every four to five years in over 45 African countries (88). The data can help policymakers identify high-risk populations and create tailored programmes to address specific needs.

### Advocate for ECC inclusion in universal health coverage

In many low- and middle-income countries, oral health services are excluded from universal health coverage (UHC) (89). Advocating for ECC treatment and prevention under UHC would expand access for all, regardless of income, and reduce the economic burden on families. An ecological study, however, suggested that the impact of health expenditure on the prevalence of global ECC was stronger than that of UHC coverage (90). The study suggests that while UHC may provide access to services, the expected reduction in ECC prevalence may not happen if the quality of dental care or preventive services is inadequate, as access does not guarantee effective care. African governments must enhance the quality of dental services within the UHC frameworks and invest in preventive care and dental health education as part of public health initiatives by allocating resources to improve health systems.

Table 2 summarises the key policies and programmes aimed at integrating ECC prevention into the broader healthcare systems. It includes details on possible sources of financial support for the initiatives, including expectations from governments, international organizations, non-governmental organizations, and private sector partnerships. Implementing these policies and programmes is expected to increase early detection of ECC, improve access to oral health services, improve hygiene practices, and reduce ECC prevalence. Challenges to implementing these proposed programmes range from infrastructure limitations and workforce shortages to funding constraints and cultural resistance.

**Table 2:** Key policies and programmes for integrating ECC prevention into the broader healthcare systems.

Proposed policy/programme	Implementation strategy	Financial support required	Expected outcome/impact	Challenges
Integrate oral health into primary healthcare systems	• Train PHC personnel to detect ECC and provide preventive care (91).	Government health budgets, WHO, UNICEF, NGOs (e.g., Bill & Melinda Gates Foundation), public-private partnerships	• Increased early detection and prevention of ECC (92).	Limited healthcare infrastructure, shortage of trained personnel, and resistance to integrating oral health into PHC.
	• Include oral health screenings in routine pediatric visits (93).		• Improved access to basic oral health services for children (94).	
	• Develop guidelines for ECC risk assessment and preventive counseling (92).		• Reduction in ECC prevalence and related disabilities (95)	
Expand community-based oral health programmes	• Partner with local NGOs, schools, and religious institutions to promote oral health awareness (96).	Local governments, international organizations (e.g., WHO, UNICEF), NGOs, and corporate social responsibility (CSR) initiatives.	• Increased community awareness of ECC prevention (97).	Limited community engagement, logistical challenges in reaching remote areas, and the sustainability of mobile clinics.
	• Deploy mobile dental clinics to remote areas for screenings, fluoride treatments, and referrals (98).		• Improved access to oral health services in underserved areas (99).	
			• Reduction in untreated ECC cases (97).	
Local production and distribution of affordable oral health products	• Provide tax breaks and subsidies for local manufacturers of oral health products (100).	Government subsidies, private sector investment, international development banks (e.g., African Development Bank), and CSR initiatives.	• Increased availability and affordability of health products (101).	Limited local manufacturing capacity, competition with imported products, and distribution challenges.
	• Establish public-private partnerships to produce and distribute affordable toothbrushes, fluoride toothpaste, and other oral hygiene products (102).		• Can improve oral hygiene practices among children and families (103).	
			• May reduce ECC prevalence.	
Implement school-based oral health education and preventive programmes	• Develop national oral health programmes focusing on daily oral hygiene, healthy eating, and ECC prevention (104).	Government education budgets, international organizations (e.g., WHO, UNICEF), NGOs, and CSR initiatives.	• Improved oral hygiene habits among school-aged children (100).	Limited funding for school health programmes, lack of parental involvement, and high dropout rates in some regions.
	• Establish regular dental screenings in schools (105, 106).		• Early detection and prevention of ECC (100, 102).	
	• Provide fluoride toothpaste and toothbrushes for daily use in schools (107).		• Reduction in ECC prevalence and related disabilities (107, 108).	
Develop public awareness campaigns	• Use mass media (radio, TV, social media) to raise awareness about ECC and its prevention (109).	Government health budgets, international organizations (e.g., WHO, UNICEF), NGOs, and CSR initiatives.	• Can increase public awareness of ECC and its impact.	Limited reach in rural areas, cultural resistance to oral health messages, and sustainability of campaigns.
	• Tailor messages to local cultural beliefs and practices (110).		• Can improve oral health-seeking behavior among families.	
	• Collaborate with community leaders to promote oral health (111).		• Can reduce untreated ECC cases.	
Strengthen oral health workforce capacity	• Train general health practitioners, pediatricians, and nurses to provide basic oral health services (112).	Government health budgets, international organizations (e.g., WHO, UNICEF), NGOs, and public-private partnerships.	• Increased availability of oral health professionals.	Limited training infrastructure, high costs of dental education, and brain drain of trained professionals.
	• Expand dental education programmes to increase the number of oral health professionals (113).		• Improved access to oral health services, especially in rural areas.	
			• Reduction in ECC prevalence and related disabilities.	
Utilise data to inform policies and programmes	• Expand surveillance systems to collect data on ECC prevalence and risk factors.	Government health budgets, international organizations (e.g., WHO, UNICEF), NGOs, and research grants.	• Can improve understanding of ECC trends and risk factors.	Limited data collection infrastructure, lack of trained personnel, and resistance to data sharing.
	• Integrate ECC monitoring into national health surveys (114).		• Can improve data-driven policy formulation and resource allocation.	
	• Establish a national ECC registry to track intervention effectiveness.		• Can ensure more effective and targeted interventions to reduce ECC prevalence.	
Advocate for ECC inclusion in universal health coverage	• Lobby governments to include ECC treatment and prevention in UHC frameworks.	Government health budgets, international organizations (e.g., WHO, UNICEF), NGOs, and public-private partnerships.	• Increased access to oral health services for all children, regardless of income (89).	Limited political will, competing health priorities, and funding constraints.
	• Advocate for increased funding for oral health services under UHC.		• Reduction in untreated ECC cases and related disabilities (115).	
			• Improved equity in oral health care (89).	

The recognition of untreated ECC as a disability provides an opportunity to leverage events like the International Day of Persons with Disabilities on December 3 to raise awareness, drive advocacy, and promote actionable interventions to eliminate untreated ECC (116–118). Framing ECC as a disabling condition highlights its profound impact on children's physical, psychological, and social well-being and positions it as a public health and social justice issue.

## Conclusion

Untreated ECC is a major public health and social justice issue in Africa, requiring urgent policy action to prevent its disabling consequences. It has an impact on the physical and psychological well-being of children in Africa, causing disability, and requiring urgent policy reforms. Successful country models demonstrate that integrating oral health into PHC, school-based programmes, and community outreach can significantly reduce ECC prevalence. Financial backing from governments, donors, partners, and private sector partnerships is critical to implementing these policies, including local production of affordable oral health products and workforce expansion. Strengthening data-driven policies through surveillance systems and advocating for ECC inclusion in the UHC can further ensure equitable access to care. By adopting these strategies, African nations can take decisive steps toward eliminating untreated ECC, improving children's well-being, and positioning oral health within broader health equity frameworks.
